# (2,9-Dimethyl-1,10-phenanthroline)(4-hydroxy­pyridine-2,6-dicarboxyl­ato)copper(II) trihydrate

**DOI:** 10.1107/S1600536809021588

**Published:** 2009-06-13

**Authors:** Janet Soleimannejad, Hossein Aghabozorg, Faranak Manteghi, Shokoh Najafi

**Affiliations:** a Faculty of Science, Department of Chemistry, Ilam University, Ilam, Iran; b Faculty of Chemistry, Islamic Azad University, North Tehran Branch, Tehran, Iran; c Department of Chemistry, Iran University of Science and Technology, Tehran, Iran

## Abstract

In the title complex, [Cu(C_7_H_3_NO_5_)(C_14_H_12_N_2_)]·3H_2_O, there are two independent neutral mol­ecules of the Cu complex along with six mol­ecules of water of hydration in the asymmetric unit. The Cu^II ^atoms in each complex adopt a distorted square-pyramidal coordination geometry being penta­coordinated by one N and two O atoms of 4-hydroxy­pyridine-2,6-dicarboxyl­ate anions and two N atoms of 2,9-dimethyl-1,10-phenanthroline (dmp) molecules. In the crystal structure, there are O—H⋯O and C—H⋯O hydrogen bonds and five π–π stacking inter­actions with centroid–centroid distances in the range 3.620 (1)–3.712 (1) Å. In addition, a C—H⋯π inter­action between a heterocyclic ring of dmp is observed to reinforce the crystal cohesion.

## Related literature

For related structures, see: Zhou *et al.* (2003 ▶, 2007 ▶); Ramos Silva *et al.* (2008 ▶); Aghabozorg, Ilaie *et al.* (2008 ▶); Aghabozorg, Manteghi & Sheshmani (2008 ▶); Aghabozorg, Motyeian, Attar Ghara­m­aleki *et al.* (2008 ▶); Aghabozorg, Motyeian, Soleiman­nejad *et al.* (2008 ▶); King *et al.* (2005 ▶); Lin *et al.* (2008 ▶).
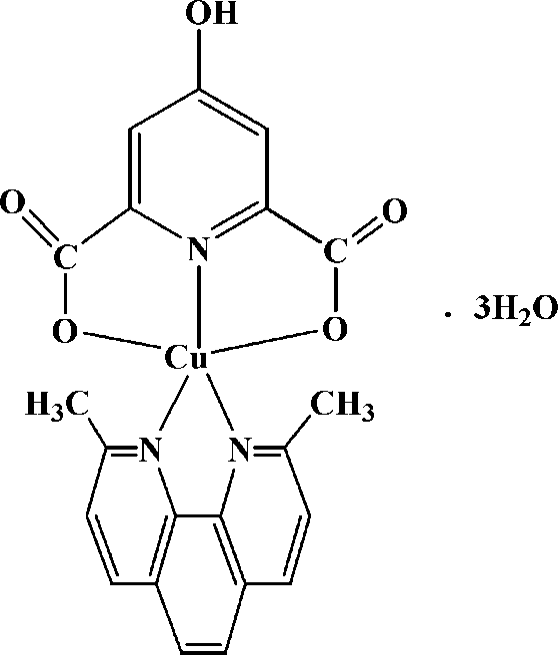


## Experimental

### 

#### Crystal data

[Cu(C_7_H_3_NO_5_)(C_14_H_12_N_2_)]·3H_2_O
*M* *_r_* = 506.95
Triclinic, 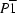

*a* = 10.0212 (2) Å
*b* = 14.8645 (3) Å
*c* = 15.4418 (3) Å
α = 91.193 (1)°
β = 106.836 (1)°
γ = 109.263 (1)°
*V* = 2061.05 (7) Å^3^
*Z* = 4
Mo *K*α radiation
μ = 1.12 mm^−1^
*T* = 150 K
0.18 × 0.18 × 0.16 mm

#### Data collection

Bruker SMART CCD area-detector diffractometer
Absorption correction: multi-scan (*SADABS*; Sheldrick, 1996 ▶) *T*_min_ = 0.824, *T*_max_ = 0.842
46562 measured reflections
12549 independent reflections
9400 reflections with *I* > 2σ(*I*)
*R*_int_ = 0.043

#### Refinement

*R*[*F* ^2^ > 2σ(*F* ^2^)] = 0.042
*wR*(*F* ^2^) = 0.127
*S* = 1.06
12549 reflections
601 parameters
H-atom parameters constrained
Δρ_max_ = 0.92 e Å^−3^
Δρ_min_ = −0.88 e Å^−3^

### 

Data collection: *SMART* (Bruker, 1998 ▶); cell refinement: *SAINT* (Bruker, 1998 ▶); data reduction: *SAINT*; program(s) used to solve structure: *SHELXS97* (Sheldrick, 2008 ▶); program(s) used to refine structure: *SHELXL97* (Sheldrick, 2008 ▶); molecular graphics: *SHELXTL* (Sheldrick, 2008 ▶); software used to prepare material for publication: *SHELXTL*.

## Supplementary Material

Crystal structure: contains datablocks I, global. DOI: 10.1107/S1600536809021588/pv2164sup1.cif

Structure factors: contains datablocks I. DOI: 10.1107/S1600536809021588/pv2164Isup2.hkl

Additional supplementary materials:  crystallographic information; 3D view; checkCIF report

## Figures and Tables

**Table 1:** Hydrogen-bond geometry (Å, °)

*D*—H⋯*A*	*D*—H	H⋯*A*	*D*⋯*A*	*D*—H⋯*A*
O3—H3*C*⋯O3*S*	0.84	1.75	2.572 (2)	166
O8—H8*C*⋯O2*S*	0.84	1.72	2.553 (2)	173
O1*S*—H1*B*⋯O9^i^	0.85	2.01	2.833 (2)	163
O1*S*—H1*A*⋯O2	0.85	2.01	2.794 (2)	153
O2*S*—H2*A*⋯O4^ii^	0.85	2.10	2.861 (2)	149
O2*S*—H2*A*⋯O7^iii^	0.85	2.64	3.136 (2)	118
O2*S*—H2*B*⋯O6*S*^ii^	0.85	1.81	2.649 (2)	168
O3*S*—H3*B*⋯O9^iv^	0.85	1.97	2.798 (2)	166
O3*S*—H3*A*⋯O5*S*	0.85	1.89	2.702 (2)	160
O4*S*—H4*A*⋯O9^v^	0.85	2.26	2.957 (3)	140
O4*S*—H4*B*⋯O2^vi^	0.85	1.97	2.811 (2)	171
O5*S*—H5*B*⋯O7^vii^	0.85	1.94	2.787 (2)	178
O5*S*—H5*A*⋯O7^viii^	0.85	2.15	2.938 (2)	154
O6*S*—H6*A*⋯O4*S*	0.85	1.95	2.764 (3)	160
O6*S*—H6*B*⋯O1*S*^ix^	0.85	2.00	2.840 (2)	170
C13—H13*B*⋯*Cg*1^x^	0.98	2.76	3.372 (2)	121

Symmetry codes: (i) 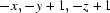; (ii) 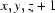; (iii) 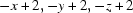; (iv) 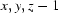; (v) 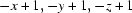; (vi) 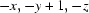; (vii) 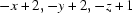; (viii) 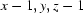; (ix) 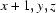; (x) 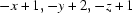. *Cg*1 is the centroid of the N4/C22–C25,C33 ring.

**Fig. 1.**
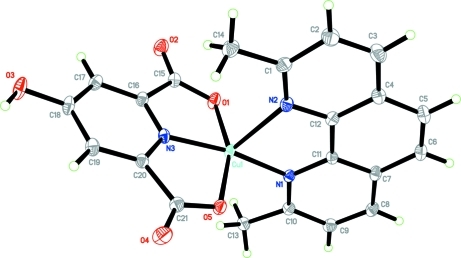
ORTEP drawing of one of the two independent molecules of [Cu(Hchel)(dmp)]. Thermal ellipsoids are shown at 50% probability level.

**Fig. 2.**
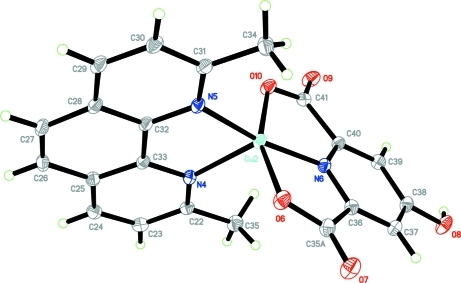
ORTEP drawing of the other independent molecule of [Cu(Hchel)(dmp)]. Thermal ellipsoids are shown at 50% probability level.

**Fig. 3.**
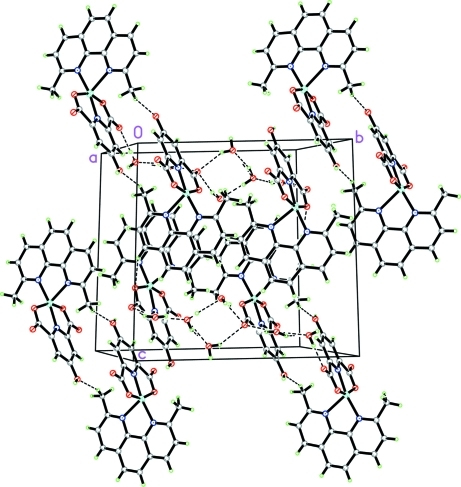
Crystal packing of the title complex, dashed lines indicate hydrogen bonds.

## supplementary crystallographic information

### Comment

There are several reports on coordination of 4-hydroxypyridine-2,6-dicarboxylic acid (H_3_chel or chelidamic acid) to metals, such as Fe(Hchel)Cl(H_2_O)_2_].(18-crown-6).2H_2_O (Zhou *et al.*, 2007), [Zn(Hchel)(H_2_O)_3_].0.25CH_3_CN.H_2_O (Zhou *et al.*, 2003), (tataH)_2_[Cu(Hchel)_2_.6H_2_O (tata: 2,4,6-triamino-1,3,5-triazine) (Ramos Silva *et al.*, 2008), (GH)_2_[Ni(Hchel)_2_].2H_2_O (G: guanidine) (Aghabozorg, Motyeian & Gharamaleki *et al.*, 2008) and {[Cd_2_(Hchel)_2_(H_2_O)_4_].4H_2_O}*_n_* (Aghabozorg, Ilaie *et al.*, 2008) [Cu(Hchel)(phen)(H_2_O)].4.5H_2_O (phen: phenanthroline) (Aghabozorg, Motyeian & Gharamaleki *et al.*, 2008; Aghabozorg, Manteghi & Sheshmani, 2008). Also, there are some reports on 2,9-dimethyl-1,10-phenanthroline (dmp) coordinated to metallic ions like [Cu(dmp)_2_][N(CN)_2_] (King *et al.*., 2005) and [Cu(HCO_2_)_2_(dmp)(H_2_O)] (Lin *et al.*, 2008). We have now prepared the title compound, (I), which contains both Hchel and dmp species coordinated to Cu^II^ atom. In this paper, we report the crystal structure of (I).

In the asymmetric unit of (I), there are two Cu-complexe molecules which slightly differ in bond lengths and bond angles (Figs. 1 and 2). However, both units have a penta-coordinated geometry around Cu^II^ atoms.

The bond angles around Cu^II^ indicate that there are two angles near linearity for each Cu^II^ atom, N3Cu1N1, 168.68 (7)°, O5Cu1O1, 159.59 (6)°, N6Cu2N5, 168.36 (7)° and O6Cu2O10, 158.49 (6)° and the other angles are close to 90°. Therefore, the coordination polyhedra of two Cu^II^ atoms will be distorted square pyramids with N2 and N4 lying on axial positions. The bonds Cu1—N2 (2.2263 (17) Å) and Cu2—N4 (2.2281 (17) Å) are significantly longer than the remaining Cu —N bonds. This can be attributed to distortion due to *d*^9^ configuration of Cu^II^ atom.

The crystal structure contains many O—H···O and C—H···O hydrogen bonds (details are in Table 1 and Fig. 3) and various π–π stacking interactions. On the other hand, the five π–π stackings have centroid-centroid distances 3.620 (1) Å (*x*,*y*,*z*), 3.712 (1) Å (*x* - 1,*y*,*z)*, 3.667 (1) Å (*x*,*y*,*z*), 3.597 (1) Å (*x* - 1,*y*,*z* - 1), 3.634 (1) Å (*x* - 1,*y*,*z*), the shortest is between N6/C36—C40 and N3/C16—C20 rings. Also, a C— H···π interaction (C13—H13B and π ring of N4/C22—C25,C33 (-*x* + 1,-*y* + 2,-*z* + 1) with distance and angle 3.372 (2) Å, 121°) is present as another interaction to the supramolecular assembly.

### Experimental

The aqeuous solution containing 2,9-dimethyl-1,10-phenanthroline (0.080 g, 0.38 mmol) and 4-hydroxypyridine-2,6-dicarboxylic acid (0.069 g, 0.38 mmol) was stirred for 15 min, then the ethanol solution of CuCl_2_.2H_2_O (0.064 g, 0.38 mmol) was carefully layered on the water solution. Blue crystals suitable for crystallography were obtained in one day.

### Refinement

All H atoms were placed geometrically and included in the refinement in riding motion approximationat with distances for methyl, aryl, hydroxyl and water hydrogen atoms being 0.98, 0.95, 0.84 and 0.85 Å, respectively, and with *U*_iso_(H) = 1.2 or 1.5*U*_eq_ of the carrier atoms.

### Crystal data

**Table:**

[Cu(C_7_H_3_NO_5_)(C_14_H_12_N_2_)]·3H_2_O	*Z* = 4
*M**_r_* = 506.95	*F*(000) = 1044
Triclinic, *P*1	*D*_x_ = 1.634 Mg m^−^^3^
Hall symbol: -P 1	Mo *K*α radiation, λ = 0.71073 Å
*a* = 10.0212 (2) Å	Cell parameters from 10056 reflections
*b* = 14.8645 (3) Å	θ = 2.3–30.3°
*c* = 15.4418 (3) Å	µ = 1.12 mm^−^^1^
α = 91.193 (1)°	*T* = 150 K
β = 106.836 (1)°	Block, blue
γ = 109.263 (1)°	0.18 × 0.18 × 0.16 mm
*V* = 2061.05 (7) Å^3^	

### Data collection

**Table:**

Bruker SMART CCD area-detector diffractometer	12549 independent reflections
Radiation source: fine-focus sealed tube	9400 reflections with *I* > 2σ(*I*)
graphite	*R*_int_ = 0.043
φ and ω scans	θ_max_ = 30.7°, θ_min_ = 1.9°
Absorption correction: multi-scan (*SADABS*; Sheldrick, 1996)	*h* = −14→13
*T*_min_ = 0.824, *T*_max_ = 0.842	*k* = −21→21
46562 measured reflections	*l* = −22→22

### Refinement

**Table:**

Refinement on *F*^2^	Primary atom site location: structure-invariant direct methods
Least-squares matrix: full	Secondary atom site location: difference Fourier map
*R*[*F*^2^ > 2σ(*F*^2^)] = 0.042	Hydrogen site location: inferred from neighbouring sites
*wR*(*F*^2^) = 0.127	H-atom parameters constrained
*S* = 1.06	*w* = 1/[σ^2^(*F*_o_^2^) + (0.0696*P*)^2^ + 0.1969*P*] where *P* = (*F*_o_^2^ + 2*F*_c_^2^)/3
12549 reflections	(Δ/σ)_max_ = 0.002
601 parameters	Δρ_max_ = 0.92 e Å^−^^3^
0 restraints	Δρ_min_ = −0.87 e Å^−^^3^

### Special details

**Table:**

Geometry. All e.s.d.'s (except the e.s.d. in the dihedral angle between two l.s. planes) are estimated using the full covariance matrix. The cell e.s.d.'s are taken into account individually in the estimation of e.s.d.'s in distances, angles and torsion angles; correlations between e.s.d.'s in cell parameters are only used when they are defined by crystal symmetry. An approximate (isotropic) treatment of cell e.s.d.'s is used for estimating e.s.d.'s involving l.s. planes.
Refinement. Refinement of *F*^2^ against ALL reflections. The weighted *R*-factor *wR* and goodness of fit *S* are based on *F*^2^, conventional *R*-factors *R* are based on *F*, with *F* set to zero for negative *F*^2^. The threshold expression of *F*^2^ > σ(*F*^2^) is used only for calculating *R*-factors(gt) *etc*. and is not relevant to the choice of reflections for refinement. *R*-factors based on *F*^2^ are statistically about twice as large as those based on *F*, and *R*- factors based on ALL data will be even larger.

### Fractional atomic coordinates and isotropic or equivalent isotropic displacement parameters (Å^2^)

**Table:**

	*x*	*y*	*z*	*U*_iso_*/*U*_eq_	
Cu1	0.18000 (3)	0.776157 (17)	0.315608 (16)	0.01236 (7)	
Cu2	0.89377 (3)	0.750300 (18)	0.759838 (16)	0.01373 (7)	
O1	−0.02604 (16)	0.67332 (11)	0.27155 (10)	0.0178 (3)	
O2	−0.17402 (17)	0.56098 (11)	0.15312 (10)	0.0217 (3)	
O3	0.15699 (18)	0.65953 (12)	−0.06028 (10)	0.0229 (3)	
H3C	0.2339	0.6929	−0.0720	0.034*	
O4	0.51539 (17)	0.92317 (11)	0.22333 (11)	0.0221 (3)	
O5	0.36783 (16)	0.88012 (10)	0.31126 (9)	0.0150 (3)	
O6	1.10226 (17)	0.84891 (11)	0.79318 (10)	0.0186 (3)	
O7	1.27334 (17)	0.95980 (12)	0.90676 (10)	0.0214 (3)	
O8	0.95793 (17)	0.90993 (11)	1.13195 (10)	0.0196 (3)	
H8C	0.8778	0.8871	1.1441	0.029*	
O9	0.57249 (17)	0.62956 (11)	0.87302 (10)	0.0206 (3)	
O10	0.70852 (16)	0.65093 (11)	0.77791 (10)	0.0169 (3)	
N1	0.17688 (18)	0.82702 (12)	0.43438 (11)	0.0115 (3)	
N2	0.28176 (19)	0.68645 (12)	0.40714 (11)	0.0139 (3)	
N3	0.16794 (19)	0.74652 (12)	0.19294 (11)	0.0128 (3)	
N4	0.75691 (19)	0.81640 (12)	0.65962 (11)	0.0140 (3)	
N5	0.89303 (19)	0.68955 (12)	0.64437 (11)	0.0133 (3)	
N6	0.91937 (19)	0.79431 (12)	0.88159 (11)	0.0125 (3)	
C1	0.3294 (2)	0.61610 (15)	0.39115 (14)	0.0163 (4)	
C2	0.3859 (3)	0.56796 (17)	0.46299 (15)	0.0220 (5)	
H2	0.4153	0.5160	0.4500	0.026*	
C3	0.3985 (3)	0.59535 (17)	0.55083 (15)	0.0220 (5)	
H3	0.4387	0.5637	0.5990	0.026*	
C4	0.3513 (2)	0.67093 (15)	0.56916 (14)	0.0158 (4)	
C5	0.3611 (2)	0.70513 (16)	0.65927 (14)	0.0191 (4)	
H5	0.4016	0.6764	0.7099	0.023*	
C6	0.3133 (2)	0.77814 (16)	0.67294 (14)	0.0174 (4)	
H6	0.3218	0.8005	0.7331	0.021*	
C7	0.2502 (2)	0.82174 (15)	0.59763 (13)	0.0151 (4)	
C8	0.1995 (2)	0.89834 (15)	0.60858 (14)	0.0164 (4)	
H8	0.2080	0.9241	0.6676	0.020*	
C9	0.1379 (2)	0.93450 (15)	0.53255 (14)	0.0164 (4)	
H9	0.1034	0.9858	0.5390	0.020*	
C10	0.1248 (2)	0.89678 (14)	0.44484 (13)	0.0133 (4)	
C11	0.2379 (2)	0.78919 (14)	0.50869 (13)	0.0125 (4)	
C12	0.2918 (2)	0.71320 (15)	0.49419 (13)	0.0137 (4)	
C13	0.0502 (2)	0.93156 (15)	0.36039 (14)	0.0154 (4)	
H13A	0.1012	0.9314	0.3151	0.023*	
H13B	0.0545	0.9970	0.3755	0.023*	
H13C	−0.0541	0.8890	0.3354	0.023*	
C14	0.3228 (3)	0.58905 (17)	0.29559 (15)	0.0211 (5)	
H14A	0.2223	0.5775	0.2542	0.032*	
H14B	0.3469	0.5306	0.2926	0.032*	
H14C	0.3948	0.6414	0.2775	0.032*	
C15	−0.0619 (2)	0.63130 (15)	0.19051 (14)	0.0153 (4)	
C16	0.0507 (2)	0.67360 (15)	0.14055 (13)	0.0141 (4)	
C17	0.0453 (2)	0.64275 (15)	0.05485 (14)	0.0162 (4)	
H17	−0.0378	0.5905	0.0178	0.019*	
C18	0.1649 (2)	0.68981 (16)	0.02292 (14)	0.0172 (4)	
C19	0.2855 (2)	0.76720 (15)	0.07962 (14)	0.0162 (4)	
H19	0.3673	0.8008	0.0595	0.019*	
C20	0.2825 (2)	0.79327 (14)	0.16487 (13)	0.0130 (4)	
C21	0.4010 (2)	0.87260 (15)	0.23753 (14)	0.0151 (4)	
C22	0.6861 (2)	0.87632 (14)	0.66901 (14)	0.0137 (4)	
C23	0.6090 (2)	0.91006 (15)	0.59242 (14)	0.0162 (4)	
H23	0.5584	0.9520	0.6004	0.019*	
C24	0.6071 (2)	0.88225 (15)	0.50639 (14)	0.0161 (4)	
H24	0.5564	0.9055	0.4549	0.019*	
C25	0.6811 (2)	0.81892 (15)	0.49529 (14)	0.0145 (4)	
C26	0.6880 (2)	0.78837 (15)	0.40854 (13)	0.0160 (4)	
H26	0.6412	0.8111	0.3553	0.019*	
C27	0.7608 (2)	0.72721 (15)	0.40165 (13)	0.0160 (4)	
H27	0.7655	0.7083	0.3438	0.019*	
C28	0.8305 (2)	0.69104 (15)	0.48090 (13)	0.0143 (4)	
C29	0.9066 (2)	0.62694 (15)	0.47755 (14)	0.0164 (4)	
H29	0.9132	0.6054	0.4211	0.020*	
C30	0.9709 (2)	0.59608 (15)	0.55649 (14)	0.0164 (4)	
H30	1.0218	0.5527	0.5545	0.020*	
C31	0.9624 (2)	0.62803 (14)	0.64040 (14)	0.0147 (4)	
C32	0.8278 (2)	0.72104 (14)	0.56680 (13)	0.0125 (4)	
C33	0.7531 (2)	0.78780 (14)	0.57459 (13)	0.0127 (4)	
C34	1.0325 (3)	0.59555 (16)	0.72729 (14)	0.0188 (4)	
H34A	1.1303	0.6440	0.7588	0.028*	
H34B	1.0442	0.5344	0.7136	0.028*	
H34C	0.9688	0.5869	0.7664	0.028*	
C35	0.6941 (3)	0.90849 (16)	0.76295 (14)	0.0191 (4)	
H35A	0.6582	0.8522	0.7931	0.029*	
H35B	0.6318	0.9480	0.7597	0.029*	
H35C	0.7974	0.9464	0.7979	0.029*	
C35A	1.1514 (2)	0.89664 (15)	0.87318 (14)	0.0157 (4)	
C36	1.0421 (2)	0.86719 (14)	0.92753 (13)	0.0133 (4)	
C37	1.0587 (2)	0.90636 (15)	1.01326 (13)	0.0146 (4)	
H37	1.1471	0.9575	1.0465	0.018*	
C38	0.9421 (2)	0.86912 (15)	1.05067 (13)	0.0147 (4)	
C39	0.8153 (2)	0.79024 (15)	1.00122 (14)	0.0145 (4)	
H39	0.7363	0.7624	1.0256	0.017*	
C40	0.8096 (2)	0.75499 (14)	0.91681 (13)	0.0128 (4)	
C41	0.6861 (2)	0.67159 (15)	0.85124 (14)	0.0155 (4)	
O1S	−0.36552 (18)	0.52690 (11)	0.25879 (11)	0.0233 (3)	
H1B	−0.4325	0.4749	0.2290	0.028*	
H1A	−0.2950	0.5259	0.2391	0.028*	
O2S	0.72446 (18)	0.85157 (12)	1.18104 (11)	0.0242 (4)	
H2A	0.6820	0.8915	1.1861	0.029*	
H2B	0.6744	0.7933	1.1823	0.029*	
O3S	0.40048 (19)	0.73524 (13)	−0.09847 (11)	0.0281 (4)	
H3B	0.4591	0.7047	−0.0971	0.034*	
H3A	0.4522	0.7936	−0.0772	0.034*	
O4S	0.3462 (3)	0.52186 (14)	0.02695 (14)	0.0551 (7)	
H4A	0.3905	0.4814	0.0346	0.066*	
H4B	0.2978	0.5028	−0.0292	0.066*	
O5S	0.55325 (19)	0.92630 (12)	−0.07300 (11)	0.0282 (4)	
H5B	0.6076	0.9614	−0.0227	0.034*	
H5A	0.4795	0.9447	−0.0935	0.034*	
O6S	0.5503 (2)	0.66922 (13)	0.16038 (13)	0.0351 (4)	
H6A	0.4896	0.6346	0.1111	0.042*	
H6B	0.5865	0.6312	0.1911	0.042*	

### Atomic displacement parameters (Å^2^)

**Table:**

	*U*^11^	*U*^22^	*U*^33^	*U*^12^	*U*^13^	*U*^23^
Cu1	0.01313 (13)	0.01541 (13)	0.00901 (11)	0.00463 (10)	0.00468 (9)	0.00021 (9)
Cu2	0.01492 (13)	0.01556 (13)	0.01061 (12)	0.00421 (10)	0.00533 (10)	−0.00019 (9)
O1	0.0158 (7)	0.0226 (8)	0.0127 (7)	0.0029 (6)	0.0059 (6)	−0.0018 (6)
O2	0.0183 (8)	0.0235 (8)	0.0176 (7)	0.0011 (7)	0.0048 (6)	−0.0016 (6)
O3	0.0210 (8)	0.0347 (10)	0.0137 (7)	0.0086 (7)	0.0080 (6)	−0.0042 (6)
O4	0.0183 (8)	0.0214 (8)	0.0247 (8)	0.0000 (6)	0.0123 (7)	−0.0027 (6)
O5	0.0160 (7)	0.0162 (7)	0.0120 (6)	0.0042 (6)	0.0051 (6)	−0.0003 (5)
O6	0.0203 (8)	0.0212 (8)	0.0140 (7)	0.0039 (6)	0.0093 (6)	−0.0007 (6)
O7	0.0144 (7)	0.0267 (9)	0.0194 (7)	0.0004 (7)	0.0081 (6)	−0.0014 (6)
O8	0.0171 (8)	0.0261 (8)	0.0135 (7)	0.0026 (7)	0.0081 (6)	−0.0056 (6)
O9	0.0188 (8)	0.0188 (8)	0.0212 (8)	−0.0010 (6)	0.0109 (7)	−0.0023 (6)
O10	0.0184 (8)	0.0164 (7)	0.0143 (7)	0.0022 (6)	0.0075 (6)	−0.0020 (6)
N1	0.0109 (8)	0.0130 (8)	0.0103 (7)	0.0031 (6)	0.0042 (6)	0.0006 (6)
N2	0.0137 (8)	0.0163 (8)	0.0127 (8)	0.0064 (7)	0.0043 (7)	0.0008 (6)
N3	0.0140 (8)	0.0163 (8)	0.0106 (7)	0.0070 (7)	0.0054 (7)	0.0029 (6)
N4	0.0169 (9)	0.0143 (8)	0.0107 (7)	0.0053 (7)	0.0045 (7)	0.0009 (6)
N5	0.0152 (8)	0.0143 (8)	0.0105 (7)	0.0050 (7)	0.0046 (7)	−0.0008 (6)
N6	0.0135 (8)	0.0137 (8)	0.0105 (7)	0.0051 (7)	0.0037 (7)	0.0011 (6)
C1	0.0146 (10)	0.0162 (10)	0.0172 (9)	0.0052 (8)	0.0042 (8)	0.0001 (8)
C2	0.0249 (12)	0.0241 (12)	0.0224 (11)	0.0168 (10)	0.0058 (9)	0.0033 (9)
C3	0.0238 (11)	0.0256 (12)	0.0205 (10)	0.0140 (10)	0.0062 (9)	0.0072 (9)
C4	0.0130 (10)	0.0190 (10)	0.0161 (9)	0.0066 (8)	0.0042 (8)	0.0031 (8)
C5	0.0174 (10)	0.0261 (12)	0.0144 (9)	0.0081 (9)	0.0049 (8)	0.0069 (8)
C6	0.0159 (10)	0.0233 (11)	0.0120 (9)	0.0060 (9)	0.0040 (8)	0.0022 (8)
C7	0.0139 (9)	0.0195 (10)	0.0114 (9)	0.0046 (8)	0.0050 (8)	0.0012 (7)
C8	0.0172 (10)	0.0187 (10)	0.0136 (9)	0.0048 (8)	0.0074 (8)	−0.0010 (8)
C9	0.0185 (10)	0.0165 (10)	0.0163 (9)	0.0068 (8)	0.0079 (8)	0.0001 (8)
C10	0.0114 (9)	0.0151 (10)	0.0137 (9)	0.0032 (8)	0.0064 (8)	0.0016 (7)
C11	0.0110 (9)	0.0140 (9)	0.0118 (8)	0.0034 (7)	0.0038 (7)	0.0025 (7)
C12	0.0120 (9)	0.0154 (10)	0.0135 (9)	0.0041 (8)	0.0048 (8)	0.0007 (7)
C13	0.0159 (10)	0.0172 (10)	0.0154 (9)	0.0077 (8)	0.0062 (8)	0.0035 (8)
C14	0.0252 (12)	0.0223 (11)	0.0191 (10)	0.0126 (10)	0.0071 (9)	−0.0003 (8)
C15	0.0146 (10)	0.0181 (10)	0.0136 (9)	0.0062 (8)	0.0044 (8)	0.0021 (8)
C16	0.0149 (10)	0.0166 (10)	0.0122 (9)	0.0072 (8)	0.0045 (8)	0.0010 (7)
C17	0.0146 (10)	0.0184 (10)	0.0136 (9)	0.0061 (8)	0.0015 (8)	−0.0015 (8)
C18	0.0188 (10)	0.0261 (11)	0.0104 (9)	0.0125 (9)	0.0048 (8)	0.0007 (8)
C19	0.0161 (10)	0.0219 (11)	0.0143 (9)	0.0093 (9)	0.0070 (8)	0.0035 (8)
C20	0.0140 (9)	0.0143 (9)	0.0119 (9)	0.0071 (8)	0.0035 (8)	0.0022 (7)
C21	0.0168 (10)	0.0156 (10)	0.0153 (9)	0.0078 (8)	0.0065 (8)	0.0014 (7)
C22	0.0129 (9)	0.0126 (9)	0.0154 (9)	0.0037 (8)	0.0051 (8)	0.0006 (7)
C23	0.0138 (10)	0.0148 (10)	0.0202 (10)	0.0050 (8)	0.0058 (8)	0.0009 (8)
C24	0.0134 (10)	0.0155 (10)	0.0171 (9)	0.0043 (8)	0.0023 (8)	0.0027 (8)
C25	0.0137 (9)	0.0145 (9)	0.0132 (9)	0.0027 (8)	0.0038 (8)	0.0016 (7)
C26	0.0159 (10)	0.0187 (10)	0.0109 (9)	0.0037 (8)	0.0034 (8)	0.0028 (7)
C27	0.0158 (10)	0.0175 (10)	0.0106 (9)	0.0014 (8)	0.0033 (8)	−0.0007 (7)
C28	0.0136 (9)	0.0141 (9)	0.0121 (9)	0.0008 (8)	0.0042 (8)	−0.0018 (7)
C29	0.0184 (10)	0.0168 (10)	0.0135 (9)	0.0039 (8)	0.0075 (8)	−0.0021 (7)
C30	0.0189 (10)	0.0165 (10)	0.0148 (9)	0.0082 (8)	0.0047 (8)	−0.0021 (8)
C31	0.0157 (10)	0.0130 (9)	0.0142 (9)	0.0034 (8)	0.0050 (8)	−0.0005 (7)
C32	0.0134 (9)	0.0125 (9)	0.0107 (8)	0.0029 (8)	0.0044 (7)	−0.0007 (7)
C33	0.0122 (9)	0.0125 (9)	0.0121 (8)	0.0024 (7)	0.0041 (7)	−0.0004 (7)
C34	0.0243 (11)	0.0219 (11)	0.0147 (9)	0.0137 (9)	0.0059 (9)	0.0026 (8)
C35	0.0225 (11)	0.0203 (11)	0.0174 (10)	0.0103 (9)	0.0075 (9)	−0.0003 (8)
C35A	0.0168 (10)	0.0193 (10)	0.0133 (9)	0.0075 (8)	0.0067 (8)	0.0013 (8)
C36	0.0141 (9)	0.0149 (9)	0.0125 (9)	0.0067 (8)	0.0049 (8)	0.0018 (7)
C37	0.0130 (9)	0.0157 (10)	0.0131 (9)	0.0040 (8)	0.0026 (8)	−0.0009 (7)
C38	0.0164 (10)	0.0178 (10)	0.0113 (9)	0.0075 (8)	0.0050 (8)	−0.0001 (7)
C39	0.0137 (9)	0.0166 (10)	0.0145 (9)	0.0047 (8)	0.0068 (8)	0.0022 (7)
C40	0.0132 (9)	0.0134 (9)	0.0121 (9)	0.0044 (8)	0.0048 (8)	0.0013 (7)
C41	0.0170 (10)	0.0136 (9)	0.0156 (9)	0.0050 (8)	0.0050 (8)	0.0017 (7)
O1S	0.0214 (8)	0.0210 (8)	0.0231 (8)	0.0006 (7)	0.0087 (7)	−0.0034 (6)
O2S	0.0240 (9)	0.0206 (8)	0.0346 (9)	0.0076 (7)	0.0189 (8)	0.0060 (7)
O3S	0.0223 (9)	0.0335 (10)	0.0307 (9)	0.0096 (8)	0.0120 (7)	−0.0012 (7)
O4S	0.0751 (17)	0.0324 (11)	0.0303 (11)	0.0164 (11)	−0.0206 (11)	−0.0035 (9)
O5S	0.0257 (9)	0.0347 (10)	0.0237 (8)	0.0149 (8)	0.0026 (7)	−0.0078 (7)
O6S	0.0297 (10)	0.0243 (9)	0.0404 (11)	0.0075 (8)	−0.0028 (8)	0.0039 (8)

### Geometric parameters (Å, °)

**Table:**

Cu1—N3	1.8959 (16)	C14—H14A	0.9800
Cu1—N1	1.9818 (16)	C14—H14B	0.9800
Cu1—O5	2.0234 (15)	C14—H14C	0.9800
Cu1—O1	2.0306 (15)	C15—C16	1.523 (3)
Cu1—N2	2.2263 (17)	C16—C17	1.372 (3)
Cu2—N6	1.8940 (16)	C17—C18	1.404 (3)
Cu2—N5	1.9787 (16)	C17—H17	0.9500
Cu2—O6	2.0257 (16)	C18—C19	1.407 (3)
Cu2—O10	2.0528 (15)	C19—C20	1.376 (3)
Cu2—N4	2.2281 (17)	C19—H19	0.9500
O1—C15	1.282 (2)	C20—C21	1.519 (3)
O2—C15	1.230 (3)	C22—C23	1.414 (3)
O3—C18	1.324 (2)	C22—C35	1.489 (3)
O3—H3C	0.8400	C23—C24	1.376 (3)
O4—C21	1.229 (3)	C23—H23	0.9500
O5—C21	1.287 (2)	C24—C25	1.412 (3)
O6—C35A	1.286 (2)	C24—H24	0.9500
O7—C35A	1.224 (3)	C25—C33	1.402 (3)
O8—C38	1.326 (2)	C25—C26	1.433 (3)
O8—H8C	0.8400	C26—C27	1.360 (3)
O9—C41	1.250 (3)	C26—H26	0.9500
O10—C41	1.267 (2)	C27—C28	1.433 (3)
N1—C10	1.331 (3)	C27—H27	0.9500
N1—C11	1.362 (2)	C28—C32	1.401 (3)
N2—C1	1.330 (3)	C28—C29	1.412 (3)
N2—C12	1.360 (3)	C29—C30	1.369 (3)
N3—C20	1.328 (3)	C29—H29	0.9500
N3—C16	1.335 (3)	C30—C31	1.406 (3)
N4—C22	1.338 (3)	C30—H30	0.9500
N4—C33	1.358 (2)	C31—C34	1.494 (3)
N5—C31	1.328 (3)	C32—C33	1.448 (3)
N5—C32	1.361 (2)	C34—H34A	0.9800
N6—C36	1.332 (3)	C34—H34B	0.9800
N6—C40	1.336 (3)	C34—H34C	0.9800
C1—C2	1.414 (3)	C35—H35A	0.9800
C1—C14	1.498 (3)	C35—H35B	0.9800
C2—C3	1.366 (3)	C35—H35C	0.9800
C2—H2	0.9500	C35A—C36	1.525 (3)
C3—C4	1.407 (3)	C36—C37	1.377 (3)
C3—H3	0.9500	C37—C38	1.408 (3)
C4—C12	1.403 (3)	C37—H37	0.9500
C4—C5	1.436 (3)	C38—C39	1.411 (3)
C5—C6	1.358 (3)	C39—C40	1.373 (3)
C5—H5	0.9500	C39—H39	0.9500
C6—C7	1.431 (3)	C40—C41	1.517 (3)
C6—H6	0.9500	O1S—H1B	0.8500
C7—C11	1.404 (3)	O1S—H1A	0.8500
C7—C8	1.417 (3)	O2S—H2A	0.8500
C8—C9	1.369 (3)	O2S—H2B	0.8500
C8—H8	0.9500	O3S—H3B	0.8500
C9—C10	1.408 (3)	O3S—H3A	0.8500
C9—H9	0.9500	O4S—H4A	0.8500
C10—C13	1.499 (3)	O4S—H4B	0.8500
C11—C12	1.442 (3)	O5S—H5B	0.8501
C13—H13A	0.9800	O5S—H5A	0.8501
C13—H13B	0.9800	O6S—H6A	0.8500
C13—H13C	0.9800	O6S—H6B	0.8501
			
N3—Cu1—N1	168.68 (7)	O1—C15—C16	114.46 (18)
N3—Cu1—O5	80.32 (7)	N3—C16—C17	120.63 (19)
N1—Cu1—O5	97.54 (6)	N3—C16—C15	111.38 (17)
N3—Cu1—O1	80.62 (7)	C17—C16—C15	127.92 (19)
N1—Cu1—O1	99.73 (6)	C16—C17—C18	118.77 (19)
O5—Cu1—O1	159.59 (6)	C16—C17—H17	120.6
N3—Cu1—N2	111.53 (7)	C18—C17—H17	120.6
N1—Cu1—N2	79.77 (6)	O3—C18—C17	117.9 (2)
O5—Cu1—N2	99.28 (6)	O3—C18—C19	123.2 (2)
O1—Cu1—N2	94.45 (6)	C17—C18—C19	118.94 (19)
N6—Cu2—N5	168.36 (7)	C20—C19—C18	118.6 (2)
N6—Cu2—O6	80.28 (7)	C20—C19—H19	120.7
N5—Cu2—O6	96.98 (7)	C18—C19—H19	120.7
N6—Cu2—O10	80.35 (7)	N3—C20—C19	120.70 (19)
N5—Cu2—O10	100.13 (7)	N3—C20—C21	111.76 (17)
O6—Cu2—O10	158.49 (6)	C19—C20—C21	127.53 (19)
N6—Cu2—N4	111.71 (7)	O4—C21—O5	125.7 (2)
N5—Cu2—N4	79.92 (7)	O4—C21—C20	120.64 (18)
O6—Cu2—N4	103.56 (6)	O5—C21—C20	113.69 (18)
O10—Cu2—N4	92.23 (6)	N4—C22—C23	121.14 (18)
C15—O1—Cu1	114.37 (13)	N4—C22—C35	117.98 (18)
C18—O3—H3C	109.5	C23—C22—C35	120.86 (18)
C21—O5—Cu1	114.81 (13)	C24—C23—C22	120.11 (19)
C35A—O6—Cu2	115.27 (13)	C24—C23—H23	119.9
C38—O8—H8C	109.5	C22—C23—H23	119.9
C41—O10—Cu2	113.04 (13)	C23—C24—C25	119.45 (19)
C10—N1—C11	119.81 (17)	C23—C24—H24	120.3
C10—N1—Cu1	123.96 (13)	C25—C24—H24	120.3
C11—N1—Cu1	116.18 (13)	C33—C25—C24	116.83 (18)
C1—N2—C12	118.95 (17)	C33—C25—C26	120.08 (18)
C1—N2—Cu1	132.37 (14)	C24—C25—C26	123.07 (18)
C12—N2—Cu1	108.69 (13)	C27—C26—C25	120.69 (19)
C20—N3—C16	122.35 (18)	C27—C26—H26	119.7
C20—N3—Cu1	118.70 (14)	C25—C26—H26	119.7
C16—N3—Cu1	118.67 (14)	C26—C27—C28	120.59 (19)
C22—N4—C33	118.74 (17)	C26—C27—H27	119.7
C22—N4—Cu2	132.73 (14)	C28—C27—H27	119.7
C33—N4—Cu2	108.52 (13)	C32—C28—C29	117.11 (18)
C31—N5—C32	120.07 (17)	C32—C28—C27	119.93 (18)
C31—N5—Cu2	123.70 (14)	C29—C28—C27	122.94 (18)
C32—N5—Cu2	115.96 (13)	C30—C29—C28	119.35 (19)
C36—N6—C40	121.98 (17)	C30—C29—H29	120.3
C36—N6—Cu2	119.09 (14)	C28—C29—H29	120.3
C40—N6—Cu2	118.81 (14)	C29—C30—C31	120.65 (19)
N2—C1—C2	120.68 (19)	C29—C30—H30	119.7
N2—C1—C14	118.75 (18)	C31—C30—H30	119.7
C2—C1—C14	120.56 (19)	N5—C31—C30	120.39 (18)
C3—C2—C1	120.7 (2)	N5—C31—C34	118.18 (18)
C3—C2—H2	119.6	C30—C31—C34	121.43 (18)
C1—C2—H2	119.6	N5—C32—C28	122.42 (18)
C2—C3—C4	119.2 (2)	N5—C32—C33	118.02 (17)
C2—C3—H3	120.4	C28—C32—C33	119.56 (18)
C4—C3—H3	120.4	N4—C33—C25	123.71 (18)
C12—C4—C3	116.91 (19)	N4—C33—C32	117.22 (17)
C12—C4—C5	119.85 (18)	C25—C33—C32	119.07 (17)
C3—C4—C5	123.25 (19)	C31—C34—H34A	109.5
C6—C5—C4	120.74 (19)	C31—C34—H34B	109.5
C6—C5—H5	119.6	H34A—C34—H34B	109.5
C4—C5—H5	119.6	C31—C34—H34C	109.5
C5—C6—C7	120.63 (19)	H34A—C34—H34C	109.5
C5—C6—H6	119.7	H34B—C34—H34C	109.5
C7—C6—H6	119.7	C22—C35—H35A	109.5
C11—C7—C8	117.47 (18)	C22—C35—H35B	109.5
C11—C7—C6	119.89 (18)	H35A—C35—H35B	109.5
C8—C7—C6	122.64 (18)	C22—C35—H35C	109.5
C9—C8—C7	118.74 (18)	H35A—C35—H35C	109.5
C9—C8—H8	120.6	H35B—C35—H35C	109.5
C7—C8—H8	120.6	O7—C35A—O6	126.1 (2)
C8—C9—C10	121.01 (19)	O7—C35A—C36	120.32 (18)
C8—C9—H9	119.5	O6—C35A—C36	113.61 (18)
C10—C9—H9	119.5	N6—C36—C37	120.64 (19)
N1—C10—C9	120.49 (18)	N6—C36—C35A	111.47 (17)
N1—C10—C13	117.63 (17)	C37—C36—C35A	127.89 (19)
C9—C10—C13	121.87 (18)	C36—C37—C38	118.71 (19)
N1—C11—C7	122.41 (18)	C36—C37—H37	120.6
N1—C11—C12	117.98 (17)	C38—C37—H37	120.6
C7—C11—C12	119.60 (18)	O8—C38—C37	117.86 (19)
N2—C12—C4	123.42 (18)	O8—C38—C39	122.96 (19)
N2—C12—C11	117.32 (17)	C37—C38—C39	119.18 (18)
C4—C12—C11	119.27 (18)	C40—C39—C38	117.95 (19)
C10—C13—H13A	109.5	C40—C39—H39	121.0
C10—C13—H13B	109.5	C38—C39—H39	121.0
H13A—C13—H13B	109.5	N6—C40—C39	121.46 (19)
C10—C13—H13C	109.5	N6—C40—C41	110.82 (17)
H13A—C13—H13C	109.5	C39—C40—C41	127.72 (19)
H13B—C13—H13C	109.5	O9—C41—O10	125.6 (2)
C1—C14—H14A	109.5	O9—C41—C40	118.47 (18)
C1—C14—H14B	109.5	O10—C41—C40	115.93 (18)
H14A—C14—H14B	109.5	H1B—O1S—H1A	99.2
C1—C14—H14C	109.5	H2A—O2S—H2B	115.2
H14A—C14—H14C	109.5	H3B—O3S—H3A	108.5
H14B—C14—H14C	109.5	H4A—O4S—H4B	96.6
O2—C15—O1	125.8 (2)	H5B—O5S—H5A	107.1
O2—C15—C16	119.72 (18)	H6A—O6S—H6B	104.7
			
N3—Cu1—O1—C15	6.39 (14)	N1—C11—C12—N2	1.9 (3)
N1—Cu1—O1—C15	174.92 (14)	C7—C11—C12—N2	−177.88 (18)
O5—Cu1—O1—C15	27.6 (3)	N1—C11—C12—C4	−178.17 (18)
N2—Cu1—O1—C15	−104.72 (15)	C7—C11—C12—C4	2.0 (3)
N3—Cu1—O5—C21	−7.58 (14)	Cu1—O1—C15—O2	172.45 (17)
N1—Cu1—O5—C21	−176.34 (14)	Cu1—O1—C15—C16	−5.7 (2)
O1—Cu1—O5—C21	−28.8 (3)	C20—N3—C16—C17	1.1 (3)
N2—Cu1—O5—C21	102.87 (14)	Cu1—N3—C16—C17	−172.68 (15)
N6—Cu2—O6—C35A	−3.25 (15)	C20—N3—C16—C15	178.32 (17)
N5—Cu2—O6—C35A	−171.81 (15)	Cu1—N3—C16—C15	4.5 (2)
O10—Cu2—O6—C35A	−29.3 (3)	O2—C15—C16—N3	−177.16 (18)
N4—Cu2—O6—C35A	106.93 (15)	O1—C15—C16—N3	1.1 (3)
N6—Cu2—O10—C41	9.37 (14)	O2—C15—C16—C17	−0.2 (3)
N5—Cu2—O10—C41	177.56 (14)	O1—C15—C16—C17	178.1 (2)
O6—Cu2—O10—C41	35.4 (2)	N3—C16—C17—C18	−0.5 (3)
N4—Cu2—O10—C41	−102.29 (14)	C15—C16—C17—C18	−177.25 (19)
N3—Cu1—N1—C10	3.2 (5)	C16—C17—C18—O3	−179.41 (19)
O5—Cu1—N1—C10	81.50 (16)	C16—C17—C18—C19	−0.3 (3)
O1—Cu1—N1—C10	−87.56 (16)	O3—C18—C19—C20	179.71 (19)
N2—Cu1—N1—C10	179.63 (17)	C17—C18—C19—C20	0.7 (3)
N3—Cu1—N1—C11	−174.1 (3)	C16—N3—C20—C19	−0.7 (3)
O5—Cu1—N1—C11	−95.83 (14)	Cu1—N3—C20—C19	173.04 (15)
O1—Cu1—N1—C11	95.11 (14)	C16—N3—C20—C21	−179.77 (17)
N2—Cu1—N1—C11	2.30 (14)	Cu1—N3—C20—C21	−6.0 (2)
N3—Cu1—N2—C1	−2.5 (2)	C18—C19—C20—N3	−0.2 (3)
N1—Cu1—N2—C1	178.3 (2)	C18—C19—C20—C21	178.70 (19)
O5—Cu1—N2—C1	−85.68 (19)	Cu1—O5—C21—O4	−174.05 (17)
O1—Cu1—N2—C1	79.16 (19)	Cu1—O5—C21—C20	6.4 (2)
N3—Cu1—N2—C12	178.01 (13)	N3—C20—C21—O4	179.77 (18)
N1—Cu1—N2—C12	−1.23 (13)	C19—C20—C21—O4	0.8 (3)
O5—Cu1—N2—C12	94.83 (14)	N3—C20—C21—O5	−0.7 (2)
O1—Cu1—N2—C12	−100.33 (14)	C19—C20—C21—O5	−179.62 (19)
N1—Cu1—N3—C20	87.4 (4)	C33—N4—C22—C23	−0.3 (3)
O5—Cu1—N3—C20	7.40 (14)	Cu2—N4—C22—C23	178.15 (15)
O1—Cu1—N3—C20	−179.94 (16)	C33—N4—C22—C35	−178.77 (18)
N2—Cu1—N3—C20	−88.83 (15)	Cu2—N4—C22—C35	−0.3 (3)
N1—Cu1—N3—C16	−98.6 (4)	N4—C22—C23—C24	−0.7 (3)
O5—Cu1—N3—C16	−178.60 (16)	C35—C22—C23—C24	177.7 (2)
O1—Cu1—N3—C16	−5.94 (15)	C22—C23—C24—C25	0.8 (3)
N2—Cu1—N3—C16	85.17 (16)	C23—C24—C25—C33	0.1 (3)
N6—Cu2—N4—C22	−3.4 (2)	C23—C24—C25—C26	−178.3 (2)
N5—Cu2—N4—C22	177.0 (2)	C33—C25—C26—C27	1.7 (3)
O6—Cu2—N4—C22	−88.16 (19)	C24—C25—C26—C27	180.0 (2)
O10—Cu2—N4—C22	77.12 (19)	C25—C26—C27—C28	0.8 (3)
N6—Cu2—N4—C33	175.18 (13)	C26—C27—C28—C32	−2.0 (3)
N5—Cu2—N4—C33	−4.43 (13)	C26—C27—C28—C29	179.6 (2)
O6—Cu2—N4—C33	90.39 (14)	C32—C28—C29—C30	1.2 (3)
O10—Cu2—N4—C33	−104.33 (13)	C27—C28—C29—C30	179.7 (2)
N6—Cu2—N5—C31	1.4 (5)	C28—C29—C30—C31	−0.4 (3)
O6—Cu2—N5—C31	76.98 (17)	C32—N5—C31—C30	0.9 (3)
O10—Cu2—N5—C31	−89.94 (17)	Cu2—N5—C31—C30	−172.86 (15)
N4—Cu2—N5—C31	179.58 (18)	C32—N5—C31—C34	179.94 (19)
N6—Cu2—N5—C32	−172.7 (3)	Cu2—N5—C31—C34	6.1 (3)
O6—Cu2—N5—C32	−97.06 (15)	C29—C30—C31—N5	−0.7 (3)
O10—Cu2—N5—C32	96.02 (15)	C29—C30—C31—C34	−179.7 (2)
N4—Cu2—N5—C32	5.54 (14)	C31—N5—C32—C28	−0.1 (3)
N5—Cu2—N6—C36	82.3 (4)	Cu2—N5—C32—C28	174.19 (15)
O6—Cu2—N6—C36	5.06 (14)	C31—N5—C32—C33	179.80 (19)
O10—Cu2—N6—C36	175.67 (16)	Cu2—N5—C32—C33	−5.9 (2)
N4—Cu2—N6—C36	−95.77 (15)	C29—C28—C32—N5	−1.0 (3)
N5—Cu2—N6—C40	−101.6 (4)	C27—C28—C32—N5	−179.53 (18)
O6—Cu2—N6—C40	−178.89 (16)	C29—C28—C32—C33	179.15 (19)
O10—Cu2—N6—C40	−8.27 (14)	C27—C28—C32—C33	0.6 (3)
N4—Cu2—N6—C40	80.28 (16)	C22—N4—C33—C25	1.2 (3)
C12—N2—C1—C2	1.7 (3)	Cu2—N4—C33—C25	−177.56 (16)
Cu1—N2—C1—C2	−177.74 (16)	C22—N4—C33—C32	−178.47 (18)
C12—N2—C1—C14	−178.02 (19)	Cu2—N4—C33—C32	2.7 (2)
Cu1—N2—C1—C14	2.5 (3)	C24—C25—C33—N4	−1.1 (3)
N2—C1—C2—C3	−2.8 (3)	C26—C25—C33—N4	177.31 (19)
C14—C1—C2—C3	176.9 (2)	C24—C25—C33—C32	178.58 (19)
C1—C2—C3—C4	1.5 (4)	C26—C25—C33—C32	−3.0 (3)
C2—C3—C4—C12	0.7 (3)	N5—C32—C33—N4	1.7 (3)
C2—C3—C4—C5	−179.6 (2)	C28—C32—C33—N4	−178.42 (18)
C12—C4—C5—C6	0.0 (3)	N5—C32—C33—C25	−178.02 (18)
C3—C4—C5—C6	−179.7 (2)	C28—C32—C33—C25	1.9 (3)
C4—C5—C6—C7	0.8 (3)	Cu2—O6—C35A—O7	−179.46 (18)
C5—C6—C7—C11	−0.2 (3)	Cu2—O6—C35A—C36	1.2 (2)
C5—C6—C7—C8	−179.7 (2)	C40—N6—C36—C37	−1.5 (3)
C11—C7—C8—C9	1.8 (3)	Cu2—N6—C36—C37	174.45 (15)
C6—C7—C8—C9	−178.7 (2)	C40—N6—C36—C35A	178.38 (17)
C7—C8—C9—C10	0.0 (3)	Cu2—N6—C36—C35A	−5.7 (2)
C11—N1—C10—C9	2.6 (3)	O7—C35A—C36—N6	−176.72 (19)
Cu1—N1—C10—C9	−174.66 (15)	O6—C35A—C36—N6	2.7 (2)
C11—N1—C10—C13	−176.24 (18)	O7—C35A—C36—C37	3.1 (3)
Cu1—N1—C10—C13	6.5 (3)	O6—C35A—C36—C37	−177.47 (19)
C8—C9—C10—N1	−2.2 (3)	N6—C36—C37—C38	−1.2 (3)
C8—C9—C10—C13	176.5 (2)	C35A—C36—C37—C38	178.94 (19)
C10—N1—C11—C7	−0.7 (3)	C36—C37—C38—O8	−177.71 (18)
Cu1—N1—C11—C7	176.72 (15)	C36—C37—C38—C39	2.9 (3)
C10—N1—C11—C12	179.48 (18)	O8—C38—C39—C40	178.69 (19)
Cu1—N1—C11—C12	−3.1 (2)	C37—C38—C39—C40	−1.9 (3)
C8—C7—C11—N1	−1.5 (3)	C36—N6—C40—C39	2.5 (3)
C6—C7—C11—N1	178.99 (19)	Cu2—N6—C40—C39	−173.44 (15)
C8—C7—C11—C12	178.31 (19)	C36—N6—C40—C41	−178.16 (17)
C6—C7—C11—C12	−1.2 (3)	Cu2—N6—C40—C41	5.9 (2)
C1—N2—C12—C4	0.6 (3)	C38—C39—C40—N6	−0.7 (3)
Cu1—N2—C12—C4	−179.87 (16)	C38—C39—C40—C41	−179.95 (19)
C1—N2—C12—C11	−179.53 (18)	Cu2—O10—C41—O9	171.00 (17)
Cu1—N2—C12—C11	0.0 (2)	Cu2—O10—C41—C40	−8.8 (2)
C3—C4—C12—N2	−1.8 (3)	N6—C40—C41—O9	−177.29 (18)
C5—C4—C12—N2	178.5 (2)	C39—C40—C41—O9	2.0 (3)
C3—C4—C12—C11	178.3 (2)	N6—C40—C41—O10	2.5 (3)
C5—C4—C12—C11	−1.4 (3)	C39—C40—C41—O10	−178.16 (19)

### Hydrogen-bond geometry (Å, °)

**Table:**

*D*—H···*A*	*D*—H	H···*A*	*D*···*A*	*D*—H···*A*
O3—H3C···O3S	0.84	1.75	2.572 (2)	166
O8—H8C···O2S	0.84	1.72	2.553 (2)	173
O1S—H1B···O9^i^	0.85	2.01	2.833 (2)	163
O1S—H1A···O2	0.85	2.01	2.794 (2)	153
O2S—H2A···O4^ii^	0.85	2.10	2.861 (2)	149
O2S—H2A···O7^iii^	0.85	2.64	3.136 (2)	118
O2S—H2B···O6S^ii^	0.85	1.81	2.649 (2)	168
O3S—H3B···O9^iv^	0.85	1.97	2.798 (2)	166
O3S—H3A···O5S	0.85	1.89	2.702 (2)	160
O4S—H4A···O9^v^	0.85	2.26	2.957 (3)	140
O4S—H4B···O2^vi^	0.85	1.97	2.811 (2)	171
O5S—H5B···O7^vii^	0.85	1.94	2.787 (2)	178
O5S—H5A···O7^viii^	0.85	2.15	2.938 (2)	154
O6S—H6A···O4S	0.85	1.95	2.764 (3)	160
O6S—H6B···O1S^ix^	0.85	2.00	2.840 (2)	170
C13—H13B···Cg1^x^	0.98	2.76	3.372 (2)	121

Symmetry codes: (i) −*x*, −*y*+1, −*z*+1; (ii) *x*, *y*, *z*+1; (iii) −*x*+2, −*y*+2, −*z*+2; (iv) *x*, *y*, *z*−1; (v) −*x*+1, −*y*+1, −*z*+1; (vi) −*x*, −*y*+1, −*z*; (vii) −*x*+2, −*y*+2, −*z*+1; (viii) *x*−1, *y*, *z*−1; (ix) *x*+1, *y*, *z*; (x) −*x*+1, −*y*+2, −*z*+1.
